# Supplementary material for: Host Mobility Drives Pathogen Competition in Spatially Structured Populations
Source: PLoS Comput Biol. 2013 Aug 15;9(8):e1003169. doi: 10.1371/journal.pcbi.1003169 (PMC3744403; doi:10.1371/journal.pcbi.1003169)
Supplement: Text S1 — The file contains the details on the model implementation, the study of the scenarios with heterogenous mobility patters and with partial cross-immunity and the discussion of the framing of the study in a realistic case. (PDF) [file pcbi.1003169.s001.pdf]

Supporting Information
Host mobility drives pathogen competition in spatially structured populations

Chiara Poletto[1,2,3,*], Sandro Meloni[4], Vittoria Colizza[2,3,5], Yamir Moreno[4,6] Alessandro Vespignani[7]
1 Computational Epidemiology Laboratory, Institute for Scientific Interchange, Turin, Italy
2 INSERM, U707, Paris, France
3 UPMC Université Paris 06, Faculté de Médecine Pierre et Marie Curie, UMR S 707, Paris, France
4 Institute for Biocomputation and Physics of Complex Systems, University of Zaragoza, Zaragoza, Spain
5 Institute for Scientific Interchange, Turin, Italy
6 Department of Theoretical Physics, University of Zaragoza, Zaragoza, Spain
7 Laboratory for the Modeling of Biological and Socio-technical Systems, Northeastern University, Boston MA, USA
∗ E-mail: chiara.poletto@isi.it

1 Implementation of the multi-strain spreading dynamics

Here we provide the details of the implementation of the multi-strain spreading dynamics.

We build the metapopulation systems by randomly generating the mobility network, as detailed in the Methods section of the main paper, and then assigning to each node an integer value for the population according to its degree, as given by Eq. (2). This guarantees the system to be at the equilibrium of the mobility dynamics in such a way that the population of each node fluctuates around the initial value for the whole duration of the simulated outbreak without any significant replenishment/depletion of individuals. As such it is consistent with a realistic situation where migration events that alter the population distribution occur at a time scale larger than the disease ones, thus for the duration of the epidemic the population is stable at the equilibrium. The epidemic is initialized on the top of the metapopulation system by extracting at random 50 subpopulations for the slow (fast) strain and moving the 0.1% of the population in the I_s (I_f) compartment, keeping the rest of the population in the susceptible compartment. We explicitly required that the two strains are not initialized within a same node to avoid interaction at the beginning of the epidemic. We tested different number of initially infected subpopulations (i.e. 10 and 25) finding the same qualitative results.

Once the system is initialized the epidemic is simulated as a discrete-time stochastic process. At each time step, corresponding to one unit of time $\Delta t \equiv 1$, both traveling and infection are simulated one after the other. For each subpopulation i traveling individuals are extracted from each of the four infection compartments by a multinomial distribution characterized by $k_i + 1$ possible outcomes which correspond to traveling in each of the k_i directions, with probability $\frac{p}{k_i}\Delta t$, and not traveling, with probability $1 - p\Delta t$. After the traveling the contagion is simulated within each node as a combination of multinomial and binomial transitions. In detail, contagion is modeled with a multinomial process to accommodate the fact that susceptible individuals can contract the infection from either one of the two strains. Transition probabilities are $\left\{\frac{\beta_f I_{i,f}}{N_i}\Delta t,\ \frac{\beta_s I_{i,s}}{N_i}\Delta t,\ 1 - \frac{\beta_f I_{i,f}+\beta_s I_{i,s}}{N_i}\Delta t\right\}$, where $\beta_f = R_0\mu$ and $\beta_s = R_0\mu/\tau$. The recovery of infectious is modeled as a binomial process with probabilities $\mu\Delta t$ and $\mu\Delta t/\tau$ for the fast and slow strain respectively. This succession of events is iterated until the epidemic gets extinct, namely in all subpopulations the I_s and I_f compartments are empty. In all simulations, except for the parameter exploration of Figure 7, we consider the scenario with $R_0 = 1.8$, $\mu = 0.6$ and $\tau = 2$.

From each run we collect the attack rate within every subpopulation produced by both the fast and

the slow strain. We perform 2000 runs for each set of parameters, collecting statistics over different realizations of the stochastic spreading dynamics as well as different initially infected nodes and network instances.

2 Heterogenous traffic distribution

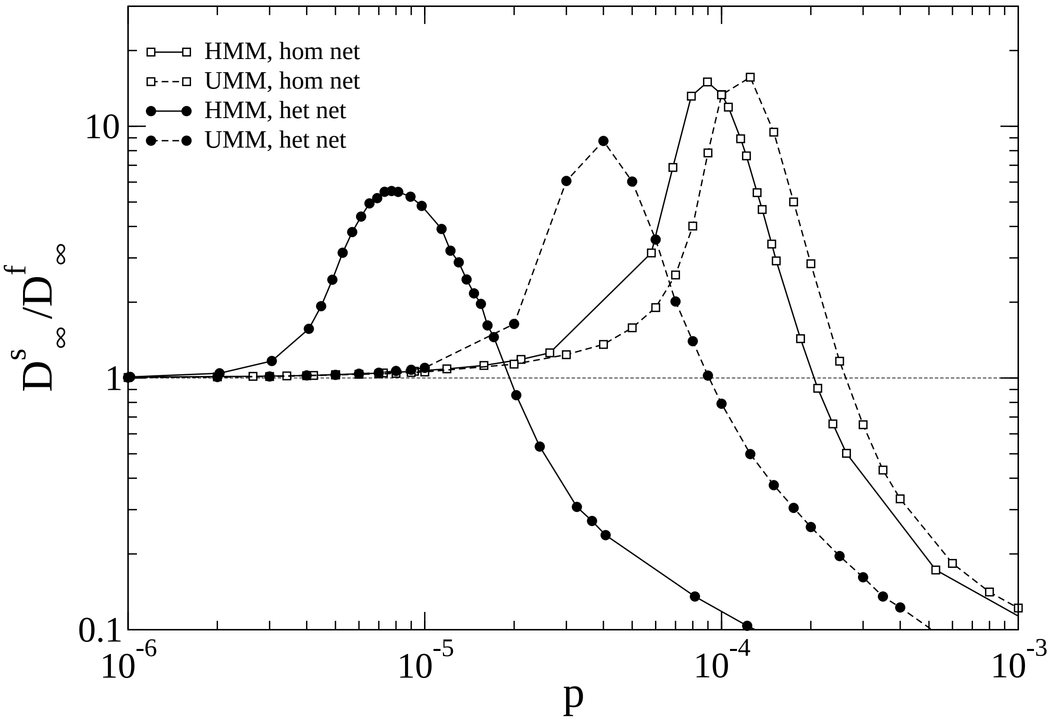

Figure S1. Competition between strains: comparison between the heterogenous mobility model (HMM) and the uniform mobility one (UMM). Ratio D^s_∞/D^f_∞ as a function of p for both homogenous and heterogenous networks and the two traffic distribution models, uniform and heterogenous. Error bars are not displayed for the sake of visualization. The networks have average degree $\bar{k} = 5$. Both strains have $R_0 = 1.8$. Other parameters are $\mu = 0.6$ and $\tau = 2$.

In this section we compare the uniform traffic distribution model considered in the main paper with the case in which the traveling probability is a heterogenous quantity defined to reproduce the statical features observed in real systems. Studies on human mobility patterns that extensively analyzed air-transportation and commuting data, have shown that the traffic along the mobility connections is a heterogenous quantity and is statistically related to the degrees of the connected nodes through a power low functional form [1, 2]. In particular for the case of the worldwide air-transportation network the following simple power law relation has been observed: $w_{kk'} = w_0(kk')^\theta$, where $w_{kk'}$ represents the

average daily number of people traveling along a link between a node with degree k and a node with degree k'. Prompted by these empirical studies we consider a heterogenous model for the traffic distribution defined as follow [3, 4]. The probability $d_{kk'}$ of traveling along a link between a node of degree k and a node of degree k' is given by

$$d_{kk'} = \frac{w_0 (kk')^\theta}{N_k} = \frac{\bar{k}\, w_0}{\bar{N}} k^{\theta-1} k'^{\theta}. \tag{S1}$$

The probability of traveling out of a node is now function of the degree,

$$p_k = k \sum_{k'} d_{kk'} P(k'|k) = \frac{w_0}{\bar{N}} \langle k^{\theta+1} \rangle k^\theta. \tag{S2}$$

where the latter equality is recovered under the assumption of uncorrelated network, i.e. $P(k'|k) = \frac{k'}{\langle k \rangle} P(k)$ ($\langle k \rangle \equiv \bar{k}$), that is fulfilled in our simulations for construction.

Figure S1 addresses the comparison between the uniform mobility model (UMM) and the heterogenous one (HMM). The figure shows the ratio D^s_∞ / D^f_∞ for the two networks considered (Poisson and power low degree distribution) and the two kinds of mobility. For the case of heterogenous traffic distribution, the parameter p represents the average value of p_k all over the network:

$$p \equiv \langle p_k \rangle = \sum_k p_k P(k) = \frac{w_0}{\bar{N}} \langle k^{\theta+1} \rangle \langle k^\theta \rangle. \tag{S3}$$

In this way we are comparing two systems with the same average traveling probability out of the nodes. According to the results of Figure S1, the multi-strain competition behavior is robust in varying the model for the traffic distribution along the links. The only difference is a shift towards lower values of p which is due to the fact that heterogeneities in the traffic distribution, analogously to heterogeneities in the network topology, increase the value of R_* and thus decrease the cross-over traveling probability p_c. This effect is more visible for the case of heterogenous network where the fluctuations introduced by the dependence between traffic and degrees are larger.

3 Partial cross-immunity

Here we present some results for the scenario in which the assumption of full cross-immunity is relaxed. Specifically, we have explored the case of partial cross-immunity between the two strains. This corresponds to consider that when an individual is infected by a given strain, he/she is fully protected against that strain, however has only a partial immunity against the other strain. We followed the multi-strain approach by Castillo-Chavez et al. [5]. Figure S2 shows what transitions are now possible under the assumption of partial cross-immunity as well as their associated transition rates. Basically, the model considers that once an individual is recovered from one strain, it acquires a degree of immunity against the other strain characterized by the parameter $1 - \sigma$, which means that with probability σ it can catch the other strain. We run numerical simulations (all the rest of parameters are the same as for the case of full cross-immunity) assuming a range of cross-immunity values compatible with those reported for influenza. Specifically, we have simulated situations in which recovered individuals from one strain may have up to 80% cross-immunity to the other strain, which roughly correspond to estimates for diverse degrees of antigenic drift of influenza [6]. This corresponds to values of σ in the range $[0, 0.2]$. As figure S3 shows, the main results reported in our paper regarding the existence of a cross-over point that depends on the mobility of hosts, are qualitatively the same within the range of σ considered. The full exploration of the role of the parameter characterizing the degree of cross-immunity in the observed competition dynamics will be the object of future work.

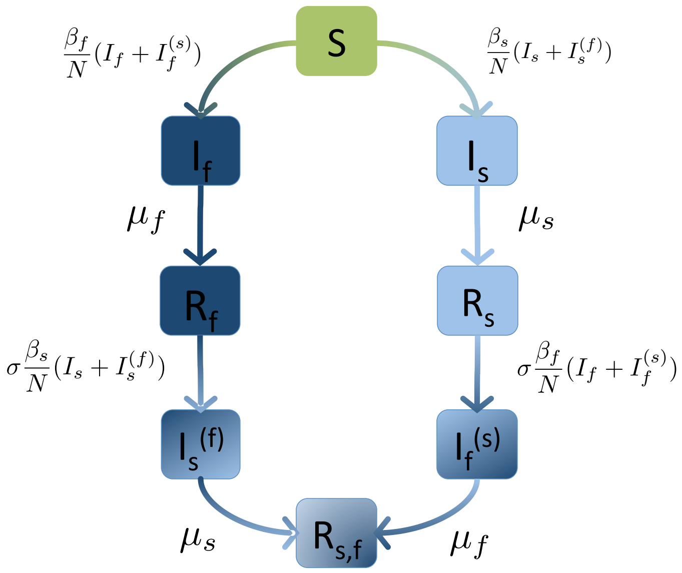

Figure S2. Partial cross-immunity setting. Schematic representation of the possible transitions when partial cross-immunity is considered. At variance with the case in which infection from one strain confers full immunity to the other strain, here we consider that with probability σ a recovered individual from one strain (slow: right branch or fast: left branch) can be infected by the other one.

4 Interpretation of the model in a real scenario

We discuss in the following the interpretation of the presented framework within the realistic setting of influenza in humans, with two strains spatially circulating through an air-transportation network. We consider the global aviation database of Ref. [7] and a timescale of 1 day. Taking into account the average daily number of people traveling on each link of the air transportation network (about 200 individuals), we obtain a corresponding traveling probability for the system under study equal to $p = 0.1$. The basic reproductive number of seasonal influenza has been estimated between $R_0 = 1.4$ and 2.0 (Ref. [8]) of the main text). Thus, referring to the case of Figure 7 of the main paper ($R_0 = 1.8$ and $\mu = 0.6$), we obtain that the air-transportation mobility scenario falls in the regime in which the fast strain is dominant for all the values of τ tested, since the cross-over traveling probability assumes values of the order of 10^{-4} for all values of τ explored. This indicates that, given the circulation of two strains characterized by total cross-immunity, and equal reproductive number and different infectious period compatible with influenza infection, the high level of mixing allowed by the modern and efficient mobility infrastructures would select the more rapidly spreading strain.

References

1. Bajardi, P., Poletto, C., Ramasco, J.J., Tizzoni, M., Colizza, V., Vespignani, A., Human Mobility Networks, Travel Restrictions, and the Global Spread of 2009 H1N1 Pandemic, PLoS ONE 6 (2011) e16591.

2. Barrat A, Barthélemy M, Pastor-Satorras R, Vespignani A (2004) The architecture of complex weighted networks. Proc Natl Acad Sci USA 101:3747–3752.

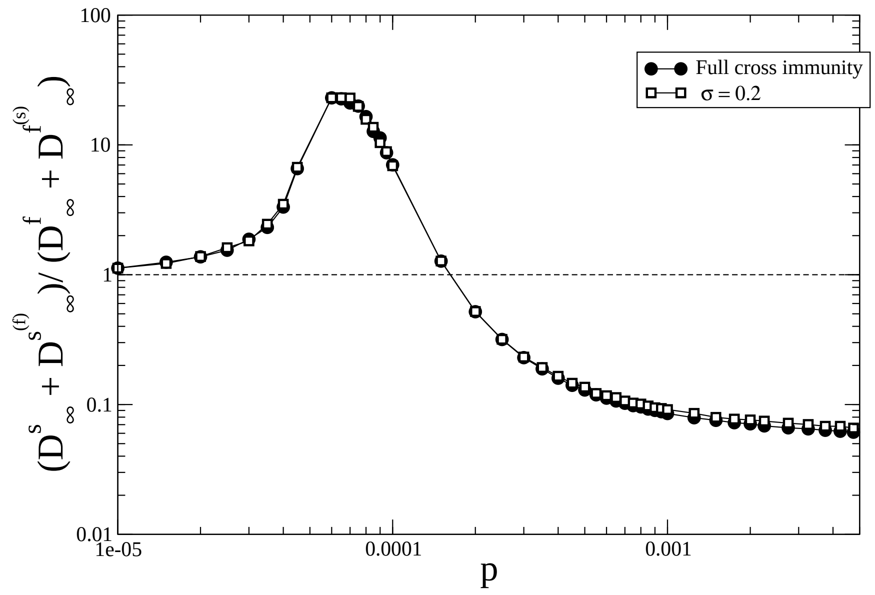

Figure S3. Ratio $\frac{D^s_\infty + D^s_\infty{}^{(f)}}{D^f_\infty + D^f_\infty{}^{(s)}}$ as a function of p for homogenous networks and two different values of σ. D^s_∞ denotes the number of populations that experience an outbreak of strain s as a primary infection (i.e., infectious individuals came directly from class S), while $D^s_\infty{}^{(f)}$ stands for the number of populations in which an outbreak of strain s takes place as a result of the re-infection of individuals already recovered from the other (fast) strain. Error bars are not displayed for the sake of visualization. The network has an average degree $\bar{k} = 5$ and both strains have $R_0 = 1.8$. Other parameters are $\mu = 0.6$ and $\tau = 2$

3. Colizza V, Vespignani A (2008) Epidemic modeling in metapopulation systems with heterogeneous coupling pattern: Theory and simulations. J Theor Biol 251:450-467.

4. Poletto C, Tizzoni M, Colizza V (2012) Heterogeneous length of stay of hosts movements and spatial epidemic spread. Scientific Reports 2:476.

5. C. Castillo-Chavez C., Hethcote H. W. , Andreasen V. , Levin S. A. , Liu W. M. (1989) Epidemiological models with age structure, proportionate mixing, and cross-immunity J. Math. Biol. 27:233-258.

6. Koelle K, Cobey S, Grenfell B, Pascual M (2006) Epochal Evolution Shapes the Phylodynamics of Interpandemic Influenza A (H3N2) in Humans. Science 314: 1898-1903.

7. Balcan D, et al. (2009) Seasonal transmission potential and activity peaks of the new influenza A(H1N1): a Monte Carlo likelihood analysis based on human mobility. BMC Med. 7:45.

8. Rohani P, Green CJ, Mantilla-Beniers NB, Grenfell BT (2003) Ecological interference between fatal diseases. Nature 422: 885-888.
